# *In vivo* fluorescence bioimaging of ascorbic acid in mice: Development of an efficient probe consisting of phthalocyanine, TEMPO, and albumin

**DOI:** 10.1038/s41598-018-19762-8

**Published:** 2018-01-24

**Authors:** Takanori Yokoi, Takayuki Otani, Kazuyuki Ishii

**Affiliations:** 1 Institute of Industrial Science, The University of Tokyo, 4-6-1 Komaba Meguro-ku, Tokyo, 153-8505 Japan; 2 Katayama Chemical Industries, Co., Ltd., 4-1-7 Ina, Minoh city, Osaka, 562-0015 Japan

## Abstract

After a groundbreaking study demonstrated that a high dose of ascorbic acid selectively kills cancer cells, the compound has been tested in the clinic against various forms of cancers, with some success. However, *in vivo* tracing of intravenously injected ascorbic acid has not been achieved. Herein, we successfully imaged ascorbic acid intravenously injected into mice based on the discovery of a novel, highly sensitive, and appropriately selective fluorescent probe consisting of silicon phthalocyanine (SiPc) and two 2,2,6,6-tetramethyl-1-piperidinyloxy (TEMPO) radicals, i.e., R2c. The radicals in this R2c were encapsulated in dimeric bovine serum albumin, and the sensitivity was >100-fold higher than those of other R2c-based probes. Ascorbic acid intravenously injected into mice was efficiently transported to the liver, heart, lung, and cholecyst. The present results provide opportunities to advance the use of ascorbic acid as cancer therapy.

## Introduction

Oxidative stress due to free radicals and reactive oxygen species is closely associated with various diseases^1^. Therefore, antioxidants are essential for reducing oxidative stress *in vivo*. Ascorbic acid, a well-known essential nutrient, i.e., vitamin C, is one such antioxidant^2,3^. Recently, ascorbic acid has also been investigated as a new class of cancer therapy, as pharmacologic concentrations (0.3–20 mM) of the compound were found to be selectively cytotoxic against cancer cells but not normal cells^4–7^. This selective toxicity is thought to be due to H_2_O_2_ produced from a high dose of ascorbic acid, as cancer cells express catalase and glutathione peroxidase less abundantly compared to normal cells^4,6–9^. Although oral doses of ascorbic acid do not generate pharmacologic concentrations in the plasma and are ineffective^10–16^, pharmacologic concentrations in the plasma are achieved by intravenous injection^17,18^. Thus, intravenous injection of ascorbic acid has been tested in the clinic against various forms of cancers, with some success. However, an *in vivo* imaging technique is needed to trace intravenously injected ascorbic acid.

Bioimaging techniques based on fluorescent probes are promising, as these modalities enable real-time observations and are highly sensitive, high-resolution, and non-destructive. For instance, various fluorophores linked to nitroxide radicals have been used as probes for ascorbic acid not only in solutions but also in biological systems^19–32^. In these probes, the nitroxide radical provides efficient fluorescence quenching^33,34^, preferably reacts with ascorbic acid, and enables quantitative determination of ascorbic acid in solutions. *In vivo* however, (1) the excitation and fluorescence (or luminescence) wavelengths should be >650 nm to penetrate deeply into living tissues, and (2) nitroxide radicals should be shielded from biological redox active species before injection of ascorbic acid, but should then efficiently react with ascorbic acid after injection. While many of these probes have been used *in vitro* and *in vivo*, the excitation wavelength of the fluorescent moiety, *e.g*., BODIPY^22–24^, rhodamine^25,26^, coumarin^27^, and others^28–31^, is nearly always ≤560 nm, which does not deeply penetrate living tissues. Similarly, luminescent probes based on redox-sensitive metal ion-modified nanomaterials^35–37^ have been reported, but their excitation or luminescence wavelengths are ≤530 nm. One exception is branched-bottlebrush polymer dual-modality organic radical contrast agents, the excitation and fluorescence wavelengths of which (~650 nm) are appropriate for deeper tissues^31^. Nevertheless, these dual-modality and other nitroxide-based luminescence probes are typically unshielded and delivered to biological systems, and the resulting fluorescent traces represent only the redox status. Therefore, these probes are generally inappropriate for imaging of ascorbic acid *in vivo* or *in vitro*, although ascorbic acid is sometimes administered to control the redox status prior to delivering nitroxide-based probes^35^.

**Figure 1:**
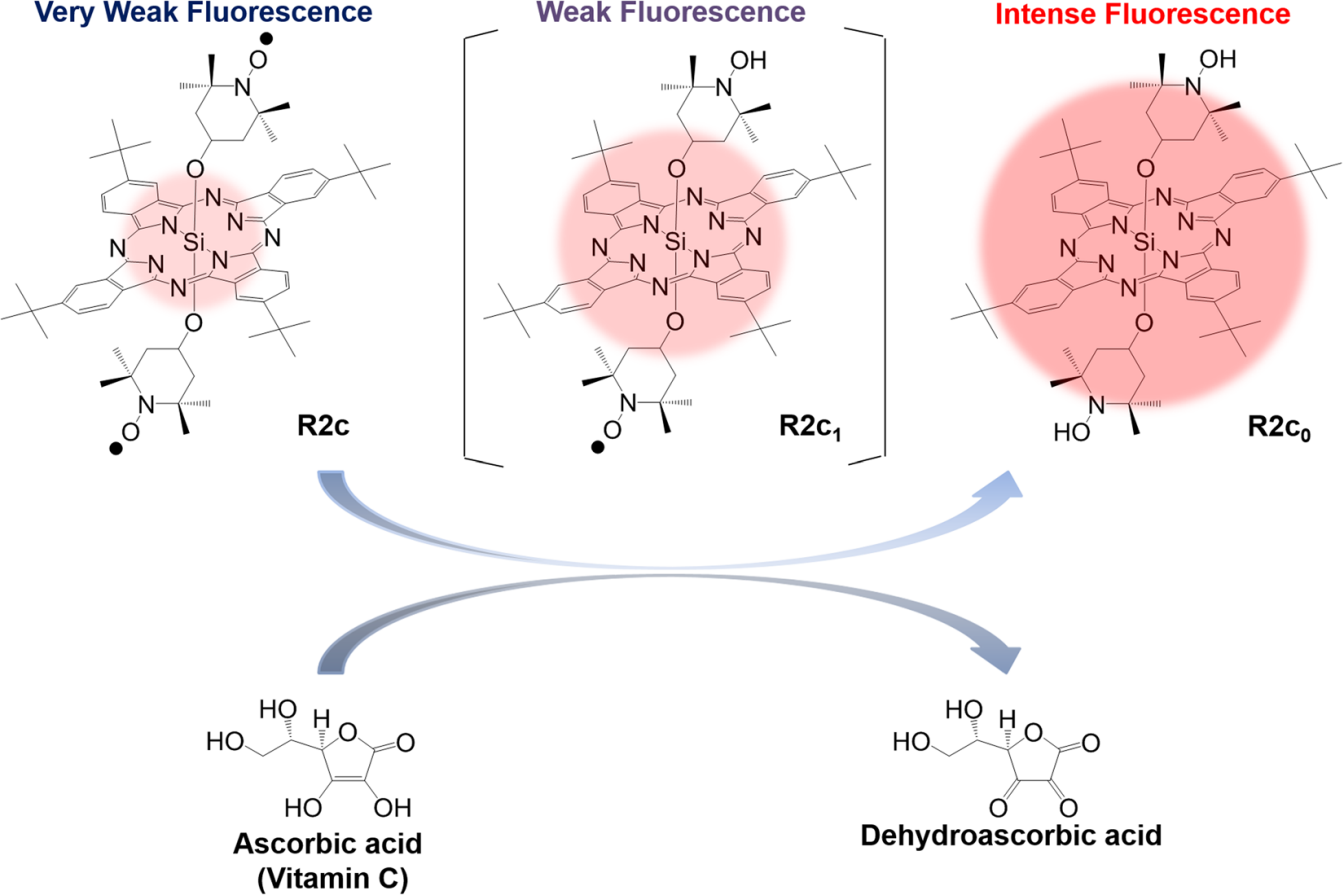
Reaction of R2c and ascorbic acid. R2c_1_ and R2c_0_ are reduced forms of R2c. Fluorescence reflects the reaction between R2c and ascorbic acid. Ascorbic acid exists as an ascorbate anion when pH is >4.2.

One alternative probe is silicon phthalocyanine (SiPc) covalently linked to two 2,2,6,6-tetramethyl-1-piperidinyloxy (TEMPO) radicals, R2c (Fig. 1 and Supplementary Fig. S1)^32^. The excitation and fluorescence wavelengths of this probe are >650 nm: the radicals of this hydrophobic probe can be shielded by encapsulation into liposomes to prevent the reaction with various biological redox active species. This probe has been used to specifically and fluorescently image ascorbic acid in cancer cells. However, lipid-based shielding deteriorated the detection limit to the mM range, and slowed the reaction rate constant to ~0.1 min^−1^ at 3 mM. Thus, a novel, shielded and highly sensitive R2c-based probe is needed.

**Figure 2:**
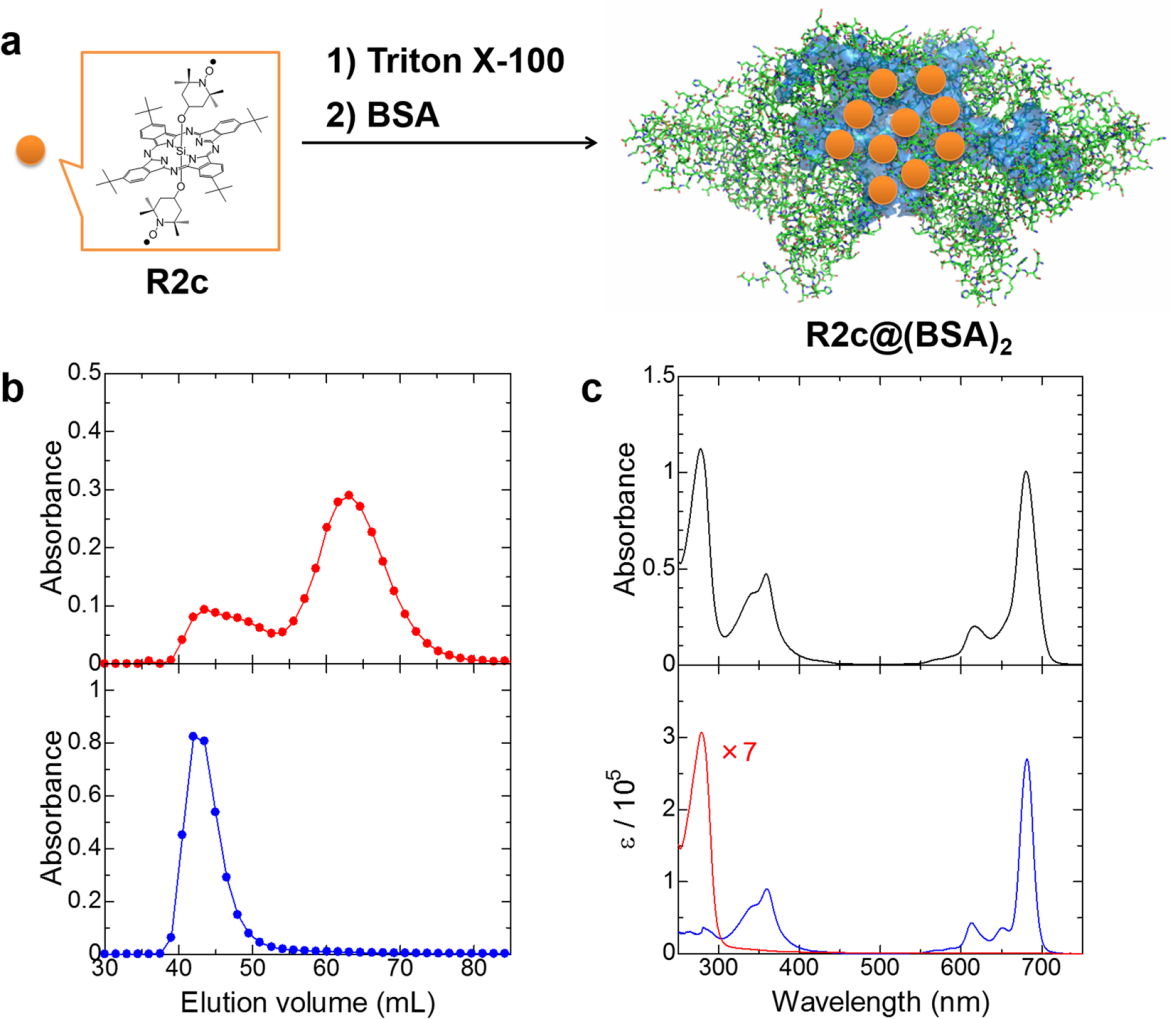
Preparation and characterization of R2c@(BSA)_2_. (**a**) Preparation of R2c@(BSA)_2_. In R2c@(BSA)_2_, dimeric BSA was estimated to contain approximately ten R2c molecules. Relatively large cavities in dimeric BSA (PDB code: 4F5S), which were calculated by PyMOL, are highlighted in blue. (**b**) Typical gel-filtration chromatographs on Sephadex G-100. Elution profiles of R2c@(BSA)_2_ (top panel: red, ~280 nm; here, absorbance of R2c at ~280 nm was removed in order to extract the absorbance of BSA, bottom panel: blue, ~680 nm). (**c**) Electronic absorption spectra of R2c@(BSA)_2_ (top panel: black), R2c (bottom panel: blue) and BSA (bottom panel: red).

We identified such a probe, consisting of R2c encapsulated in bovine serum albumin (BSA, Fig. 2a). This probe was serendipitously discovered when the activity of R2c encapsulated in Triton X-100 (R2c@TX-100) was evaluated in serum. In this experiment, R2c was unexpectedly found to be selectively encapsulated into dimeric BSA: the formed R2c@(BSA)_2_ complex was reacted with ascorbic acid with high sensitivity and appropriate selectivity. In this study, we characterized the R2c@(BSA)_2_ probe *in vitro* and *in vivo*.

## Results

### Preparation and characterization of R2c@(BSA)_2_

R2c@(BSA)_2_ was prepared by adding an aqueous solution of BSA to an aqueous solution of R2c@TX-100, followed by ultrafiltration for purification. The complex was then analysed several times by gel-filtration chromatography on a Sephadex G-100 column, as typically shown in Fig. 2b. BSA, monitored at ~280 nm, eluted in two peaks at 63 and 43 mL, which agrees well with native BSA^38,39^. These peaks were attributed to BSA and its dimer, respectively: the ratio of elution volumes ranging from 1.3 to 1.5 in several experiments is consistent with the results of a previous study^39^. On the other hand, R2c, which was selectively monitored at ~680 nm, eluted in only one peak that overlapped with dimeric BSA at 43 mL. Additionally, the elution profile of R2c@(BSA)_2_ clearly differed from that of its starting material, i.e., R2c@TX-100, and electronic absorption spectra were distinguishable between R2c@(BSA)_2_ and R2c@TX-100 eluted from the column. These results indicate that R2c is transferred from the micelle of Triton X-100 to dimeric BSA but not encapsulated into monomeric BSA.

A sharp, intense Q-absorption band was observed on the electronic absorption spectrum of R2c@(BSA)_2_, as shown in Fig. 2c. This result indicates that R2c is monomeric, and that the two bulky, axial TEMPO ligands prevent SiPc aggregation that would quench fluorescence. These features are an additional advantage over other fluorescent probes. Finally, the ratio of R2c to dimeric BSA was estimated to be approximately 10 based on the Q absorption band at 680 nm and circular dichroism at 220 nm which are directly attributable to R2c and BSA, respectively.

R2c radicals encapsulated in dimeric BSA or Triton X-100 were characterized by electron spin resonance spectroscopy in aqueous media (see Supplementary Fig. S2). The spectra of both complexes showed broad peaks and were obviously different from the isotropic spectrum observed in toluene at room temperature^33,41^, but were similar to the anisotropic spectrum observed in frozen toluene^40^. These results indicate that R2c radicals tumble freely in toluene, but tumble much more slowly in dimeric BSA and Triton X-100, confirming that the radicals are encapsulated in large molecules with low rotational mobility, and are relatively immobile at the binding site.

**Figure 3:**
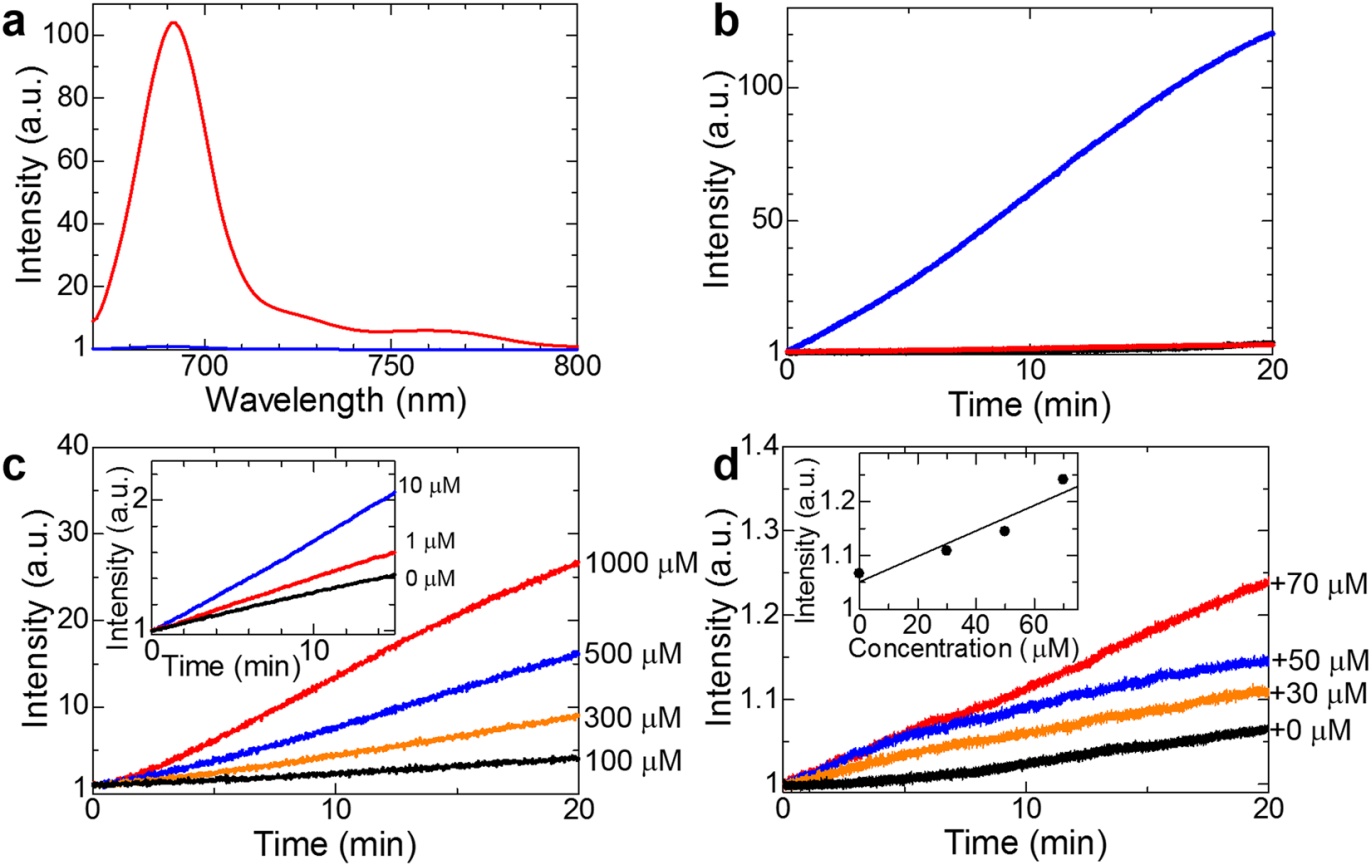
Fluorescence detection of ascorbic acid. (**a**) Fluorescence spectra of R2c@(BSA)_2_ in the presence (red) or absence (blue) of 5 mM ascorbic acid at pH 3 (λ_ex_ = 650 nm). (**b**) Time courses of fluorescence from R2c@(BSA)_2_ (blue line), R2c@TX-100 (black line), and liposomal R2c (red line) at pH 7.4, after the addition of ascorbic acid (final conc.: 10 mM). (**c**) Time courses of R2c@(BSA)_2_ fluorescence in aqueous solutions at pH 3 after addition of 100 µM (black), 300 µM (orange), 500 µM (blue), and 1000 μM (red) ascorbic acid. Inset, time courses for 0 µM (black), 1 µM (red), and 10 µM (blue) ascorbic acid. The fluorescence change in the absence of ascorbic acid was much smaller than the maximum fluorescence change after the ascorbic acid addition, and thus, R2c@(BSA)_2_ was sufficiently stable for these experiments. (**d**) Time courses of R2c@(BSA)_2_ fluorescence in aqueous solutions at pH 3 after addition of bovine sera supplemented with 0 µM (black), 30 µM (orange), 50 µM (blue), and 70 µM (red) ascorbic acid. Inset, dependence of fluorescence intensity at 20 min on ascorbic acid concentration.

Representative fluorescence spectra of R2c@(BSA)_2_ before and after exposure to ascorbic acid are shown in Fig. 3a. Fluorescence due to SiPc at around 690 nm was weak in the absence of ascorbic acid, as TEMPO radicals change the S_1_ → T_1_ intersystem crossing to the S_n_′ → S_1_′, T_n_′ → T_1_′, and T_n_′ → T_2_′ transitions, the spin multiplicities of which were identical between the initial and final states (see Supplementary Fig. S1)^32,33,40–43^. In contrast, fluorescence increased in the presence of ascorbic acid, which reacts with TEMPO radicals. Indeed, fluorescence from liposomal, BSA-encapsulated, and detergent-encapsulated R2c increased over time at pH 7.4 after adding ascorbic acid (final conc.: 10 mM), although, the fluorescence increase was more rapid and intense in R2c@(BSA)_2_ than in liposomal R2c and R2c@TX-100 (Fig. 3b). Furthermore, the time courses of R2c@(BSA)_2_ fluorescence were sigmoidal and strongly dependent on pH (see Supplementary Figs S3 and S4).

The reaction between R2c and ascorbic acid (AA) can be approximately modelled as the consecutive reaction, ${\rm{R2c}}\,\mathop{\longrightarrow }\limits^{{\rm{A}}{\rm{A}}}\,{{\rm{R2c}}}_{1}\mathop{\longrightarrow }\limits^{{\rm{A}}{\rm{A}}}\,{{\rm{R2c}}}_{0}$, where the first and second rate constants are *k*_1_ and *k*_2_, respectively. By solving simultaneous differential equations on the assumption that [AA] is constant, R2c@(BSA)_2_ fluorescence over time, F(*t*), can be calculated as the sum of fluorescence from the reactant R2c and the products R2c_1_ and R2c_0_ (Fig. 1 and Supplementary Fig. S1):

1 $$\begin{array}{lll}{\rm{F}}({\rm{t}}) & \propto  & {{\rm{\Phi }}}_{{\rm{F}}}^{{\rm{R}}{\rm{2}}{\rm{c}}}\,[{\rm{R}}{\rm{2}}{\rm{c}}]+{{\rm{\Phi }}}_{{\rm{F}}}^{{\rm{R}}{\rm{2}}{{\rm{c}}}_{1}}\,[{\rm{R}}{\rm{2}}{{\rm{c}}}_{1}]+{{\rm{\Phi }}}_{{\rm{F}}}^{{\rm{R}}2{{\rm{c}}}_{0}}\,[{\rm{R}}2{{\rm{c}}}_{0}]\\  & = & {[{\rm{R}}2{\rm{c}}]}_{0}[{{\rm{\Phi }}}_{{\rm{F}}}^{{\rm{R}}2{{\rm{c}}}_{0}}+\frac{1}{{{k}}_{{1}}-{{k}}_{{2}}}\{({{\rm{\Phi }}}_{{\rm{F}}}^{{\rm{R}}2{\rm{c}}}{{k}}_{{1}}-{{\rm{\Phi }}}_{{\rm{F}}}^{{\rm{R}}2{\rm{c}}}{{k}}_{{2}}-{{\rm{\Phi }}}_{{\rm{F}}}^{{\rm{R}}2{{\rm{c}}}_{1}}{{k}}_{{1}}\\  &  & +\,{{\rm{\Phi }}}_{{\rm{F}}}^{{\rm{R}}2{{\rm{c}}}_{0}}{{k}}_{{2}})\exp (-{{k}}_{{1}}[{\rm{AA}}]{t})\\  &  & +\,({{\rm{\Phi }}}_{{\rm{F}}}^{{\rm{R}}2{{\rm{c}}}_{1}}-{{\rm{\Phi }}}_{{\rm{F}}}^{{\rm{R}}2{{\rm{c}}}_{0}}){{k}}_{{1}}\exp (-{{k}}_{{2}}[{\rm{AA}}]{t})\}]\end{array}$$

Here, ${{\rm{\Phi }}}_{{\rm{F}}}^{{\rm{R2c}}}$, ${{\rm{\Phi }}}_{{\rm{F}}}^{{{\rm{R2c}}}_{{\rm{1}}}}$, and ${{\rm{\Phi }}}_{{\rm{F}}}^{{{\rm{R2c}}}_{{\rm{0}}}}$ denote the Φ_F_ values of R2c, R2c_1_, and R2c_0_, respectively. Equation 1 reproduces the fluorescence time course of R2c@(BSA)_2_ very well, as shown in a representative case in Supplementary Fig. S3b. This time course is very different from that of liposomal R2c, which could be reproduced by a single exponential function, as the rate-determining step is the capture of ascorbic acid into liposomes^32^. Accordingly, the differences in the time course indicate that the invasive process is negligible for R2c@(BSA)_2_.

Collectively, the data indicate that R2c is encapsulated in dimeric BSA and is in a chemically different environment than in Triton X-100 or liposomes.

### Detection of ascorbic acid in aqueous solutions

To determine the detection limit, fluorescence was measured over time in aqueous solutions supplemented with different concentrations of ascorbic acid (Fig. 3c). In this experiment, fluorescence was measured at pH 3 to maximize intensity (see Supplementary Fig. S4). Surprisingly, the limit of detection was determined to be ~1 μM for R2c@(BSA)_2_, indicating that the sensitivity was improved by more than 100-fold compared to the limit of detection (0.1 mM) for liposomal R2c. In contrast to the high reactivity for ascorbic acid, the fluorescence intensity of R2c@(BSA)_2_ was only slightly influenced by the addition of typical biological redox species, such as glutathione or hydrogen peroxide. This indicates that R2c@(BSA)_2_ should be a useful fluorescence probe for detecting ascorbic acid in biological environments.

In bovine or human serum, a model of circulating blood, although various redox species naturally existing in serum react with ascorbic acid and reduce the fluorescence increases, the fluorescence intensity of R2c@(BSA)_2_ exhibited a linear relationship with the amount of supplemented ascorbic acid (0, 30, 50 and 70 μM, Fig. 3d). Because the concentration of ascorbic acid is thought to be ~50 μM in normal human blood^17,18^, and because the excitation and fluorescence wavelengths of the SiPc fluorophore are in the red area of the spectrum which can penetrate red blood, R2c@(BSA)_2_ may enable quantification of ascorbic acid in the human blood.

### Fluorescence imaging of ascorbic acid in mice

**Figure 4:**
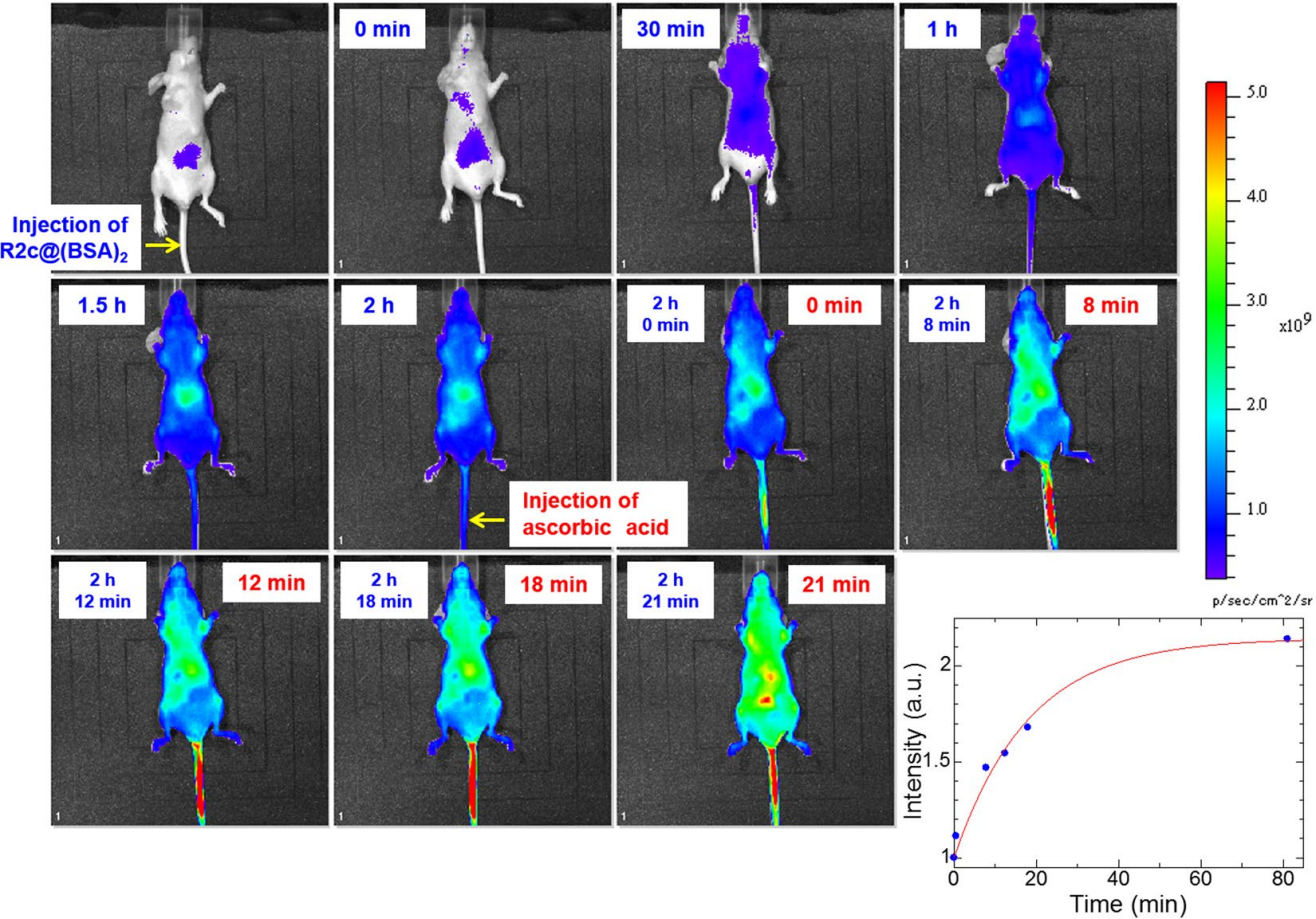
Fluorescence imaging of a mouse injected with R2c@(BSA)_2_ and ascorbic acid. Representative *in vivo* R2c@(BSA)_2_-based fluorescence imaging of intravenously injected ascorbic acid in a mouse, and time course of *in vivo* fluorescence after ascorbic acid injection (blue dots in right bottom). Mice were injected with 125 μL phosphate buffered saline containing 118 μM R2c@(BSA)_2_ via the tail vein (0.7 nmol per gram of body weight). Subsequently, a total dose of 0.23 mg of ascorbic acid per gram of body weight was administered to mice by intravenously injecting 50 μL phosphate-buffered saline containing 512 mM ascorbic acid 2 h after initial injection of the fluorescent probe. Time elapsed after R2c@(BSA)_2_ injection is indicated at the upper left in each image, while that after ascorbic acid injection is shown at the upper right in each image. The time course can be analysed by a single exponential function (red line).

**Figure 5:**
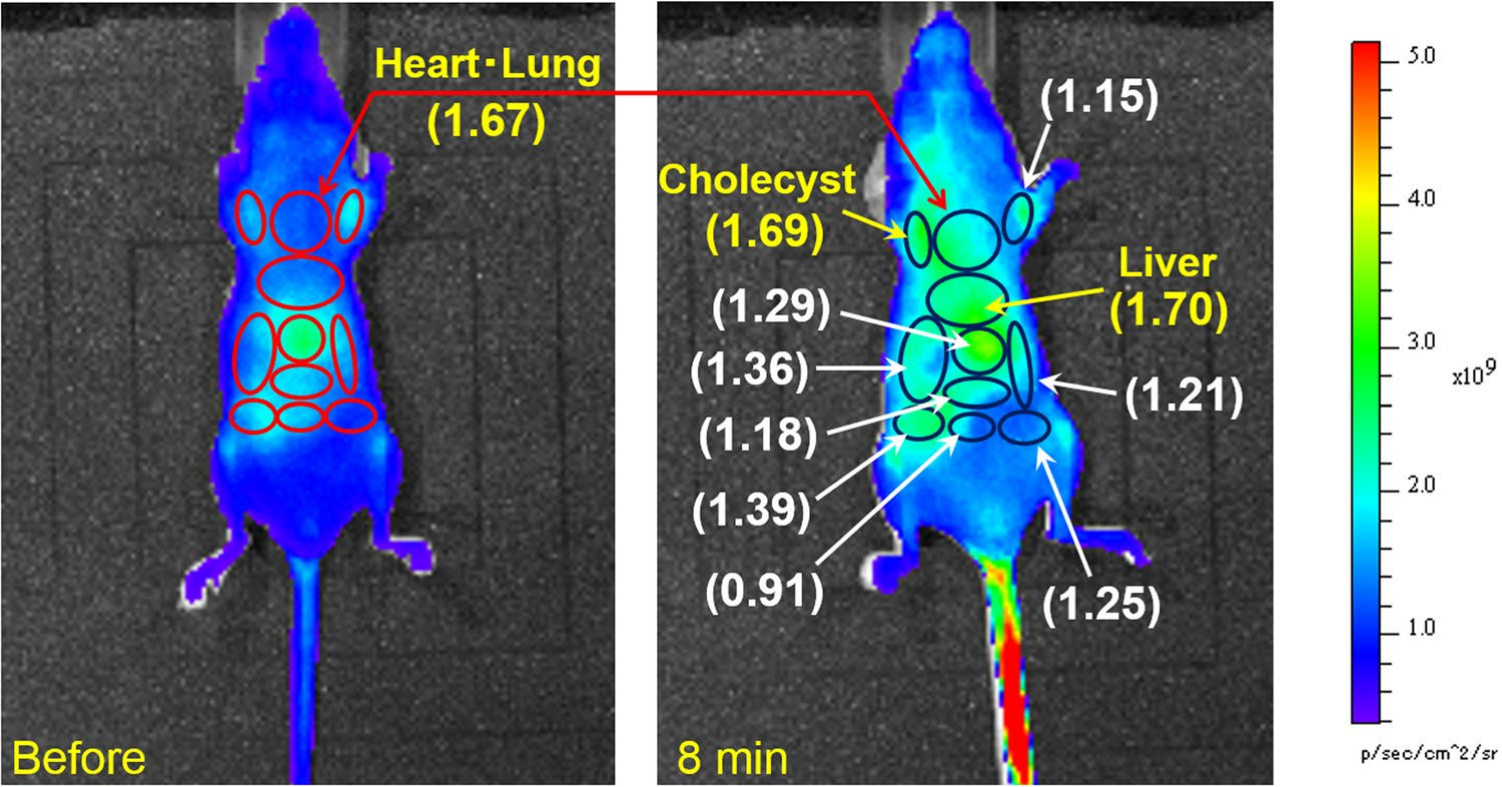
Ratio of fluorescence intensity of imaging. Ratios were calculated using fluorescence intensities before and after ascorbic acid injection (8 min).

We then tested R2c@(BSA)_2_ as a fluorescent probe to trace ascorbic acid intravenously injected into mice (Fig. 4). Initial injection of R2c@(BSA)_2_ via the tail vein induced red fluorescence within 30 min due to ascorbic acid in the blood of mice. Within 1 h, the entire body had become uniformly fluorescent without notable localized punctae, indicating that the probe had circulated over the whole body of the mouse via the blood vessels. Two hours after the initial injection of R2c@(BSA)_2_, subsequent injection of ascorbic acid (0.23 mg of ascorbic acid per gram of body weight) instantly increased the fluorescence within 10 min not in the tail vein but around the abdomen (Fig. 4), strictly capturing the reaction between R2c@(BSA)_2_ and exogenous ascorbic acid transported from the tail vein to the abdomen. This instant fluorescence increase resembles the results of previous pharmacokinetics studies, in which the ascorbic acid concentration reached to peak plasma concentrations at several minutes in rats^6^. The fluorescent region gradually expanded from the abdomen and spread throughout the body within 1 h. Overall, total fluorescence from the whole body increased exponentially after intravenous injection of ascorbic acid (Fig. 4). Several tens of minutes after ascorbic acid injection, the fluorescence intensity at the organs, such as the heart, lung and liver, had doubled, and thus, the fluorescence increase over time, which depends on both the diffusion of R2c@(BSA)_2_ and reaction with ascorbic acid in the blood, was approximated to be several tens of minutes. These changes were not observed in mice injected with R2c@(BSA)_2_ but not ascorbic acid (see Supplementary Fig. S5). Further, concentrations of ascorbic acid were examined using a Vitamin C Assay Kit: 15–20 min after injecting ascorbic acid, the concentrations were determined to be 0.15 mg/g in the liver and 0.1 mg/g (0.6 mM) in the plasma from the vena cava. Thus, we successfully imaged intravenously injected ascorbic acid in mice for the first time. We note that the changes in fluorescence before and after injection of ascorbic acid were dependent on the organs, as shown in Fig. 5. Eight minutes after ascorbic acid injection, the ratios of fluorescence intensity significantly increased at several organs, such as the liver, heart, lung, and cholecyst.

**Figure 6:**
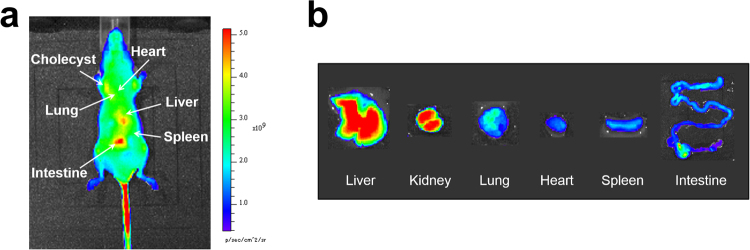
*In vivo* and *ex vivo* imaging of a mouse injected with R2c@(BSA)_2_ and ascorbic acid. (**a**) *In vivo* fluorescence imaging of R2c@(BSA)_2_ in a mouse 81 min after ascorbic acid injection. (**b**) Fluorescence imaging of excised liver, kidney, lung, heart, spleen, and intestine.

Because fluorescence imaging of deeper animal tissues is often underestimated due to optical interference from other tissues, organs were excised 1 h after intravenous injection of ascorbic acid and imaged *ex vivo* (Fig. 6). Intense fluorescence was observed in the liver, kidney, and intestine. Both *in vivo* and *ex vivo* results were reproducible, demonstrating that *in vivo* fluorescence imaging accurately captures fluorescence from the organs.

Liposomal R2c was also tested in similar experiments (see Supplementary Fig. S7), and was found to accumulate in the liver. The fluorescence increase from liposomal R2c was smaller than that of R2c@(BSA)_2_, in agreement with *in vitro* measurements. Taken together, the data indicate that R2c@(BSA)_2_ is an appropriate fluorescent probe for imaging ascorbic acid *in vivo*.

## Discussion

We successfully imaged ascorbic acid intravenously injected into mice. In addition to efficient fluorescence quenching because of spin exchange in R2c, shielding of TEMPO radicals was a key factor. Shielding was achieved by encapsulation into the hydrophobic cavity in dimeric BSA, appropriately preventing TEMPO radicals from redox reactions with natural biological redox active species even *in vivo* (Fig. 4). This approach differs from those in previous reports describing unshielded nitroxide radical spin probes^22–31^.

Notably, ascorbic acid elicited more intense fluorescence from R2c@(BSA)_2_ than from liposomal R2c. The sensitivity of liposomal R2c is lower because leaching of ascorbic acid into liposomes, a rare event, is the rate-determining step^32^. In contrast, R2c@(BSA)_2_ fluorescence can be modelled as the consecutive reaction of the two TEMPO radicals in R2c, indicating that dimeric BSA shields TEMPO radicals without significantly impeding reactions with ascorbic acid.

The hydrophobic cavity encapsulating R2c is formed at the interface between two BSA molecules, as illustrated in Fig. 2a. This interface is sufficiently large and appropriate to accommodate bulky R2c molecules containing substituents not only at peripheral positions but also at axial positions. In contrast, the hydrophobic cavity in monomeric BSA would be too small and inappropriate. Gel-filtration chromatography confirmed that the complex between R2c and dimeric BSA was selectively formed. Further, the hydrophobic cavity at the intermolecular boundary was likely to be structurally flexible, which is probably a key feature enabling both moderate shielding from biological redox reactions and high reactivity with ascorbic acid.

R2c@(BSA)_2_ dispersed throughout the mouse body without strong localized accumulation: this was another advantageous feature that enabled detailed *in vivo* tracing of intravenously injected ascorbic acid. Prior to ascorbic acid injection, the fluorescence at each organ should be proportional to the amount of R2c@(BSA)_2_ that reacted with naturally existing ascorbic acid at that organ. On the other hand, fluorescence after ascorbic acid injection should be proportional to the amount of R2c@(BSA)_2_ that reacted with exogenous ascorbic acid, which contains not only the local reaction of R2c@(BSA)_2_ at the corresponding organ but also the reacted R2c@(BSA)_2_ diffused from other organs. Thus, the gradual increase in fluorescence at each organ may include reacted R2c@(BSA)_2_ that had simply diffused from other organs, and therefore, it is challenging to distinguish at this stage between fluorescence due to local reactions at the organ and fluorescence due to diffusion from other organs. However, the immediate increase in fluorescence (within several mininutes after ascorbic acid injection) around several organs, i.e., the liver, heart, lung, and cholecyst, could reasonably be attributed to the local reactions at the corresponding organs, as the diffusion time of R2c@(BSA)_2_ from other organs was approximated to be several tens of minutes in mice. Thus, this result indicates that intravenously injected ascorbic acid is efficiently transported to the liver, heart, lung, and cholecyst.

In summary, we demonstrated that R2c encapsulated in dimeric BSA is appropriate for fluorescence imaging of ascorbic acid *in vivo* in mice. Although electron spin resonance-computed tomography remains useful for monitoring the *in vivo* redox status based on nitroxide radicals^44–48^, fluorescence imaging based on R2c@(BSA)_2_ is advantageous because of its high sensitivity, high resolution, and non-destructiveness. Importantly, imaging of injected ascorbic acid may help advance its use as cancer therapy. Finally, the newly discovered ability of dimeric BSA to provide a sensitive but selective environment for fluorescent probes should provide new opportunities for developing other probes for chemical biology.

## Methods

### Synthesis of R2c

R2c was synthesized as previously described^41^. (Dihydroxy)SiPc (0.013 mmol, Aldrich Co.) and 4-hydroxy-TEMPO (1.3 mmol, Tokyo Chemical Industry Co., Ltd.) were refluxed in toluene (65 mL) for 2 days. After basic alumina and gel permeation (Bio-Beads SX1, Bio-Rad) chromatography, R2c was isolated.

### Preparation of R2c@TX-100

Briefly, 2 mL diluted aqueous solution of Triton X-100 (Kanto Chemical Co., water:Triton X-100, 50:1, v/v) was added to 0.7 mg R2c and 500 mg glass beads, which was sonicated for 30 min.

### Preparation of R2c@(BSA)_2_

BSA, in which fatty acids were depleted, was purchased from Aldrich Co., and its purity is ≥96%. Next, 2 mL aqueous solution of R2c@TX-100 was stirred for 3 h with 100 mg BSA. The product was purified by ultrafiltration at molecular weight cut-off 10,000 Da, followed by membrane filtration (pore size: 0.45 μm).

### Gel-filtration chromatography

Samples were loaded at 0.1–0.3 mL min^−1^ on a Sephadex G-100 column (internal diameter: 2.1 or 2.4 cm, height: 27 or 21 cm) in 0.75 M Tris-HCl buffer with 1 M NaCl. Electronic absorption spectra of every fraction were recorded with a JASCO V-570 spectrometer.

### Fluorescence spectroscopy

A 650 nm diode laser (LDX Optronics LDX-2515-650) with a mechanical chopper was used as excitation source. Fluorescence was measured using a monochromator (JASCO CT-25CP) and a photomultiplier (Hamamatsu Photonics R928). Time courses obtained through a lock-in amplifier (Stanford Research SR830) were recorded using a digital oscilloscope (Iwatsu-LeCroy LT342).

### Fluorescence measurements in aqueous solutions

R2c@(BSA)_2_ fluorescence was measured using a 10 mm optical cuvette. The pH values of the following aqueous solutions were maintained at pH 2–8 using phosphate buffer. To detect ascorbic acid in aqueous solutions, 0.2 mL aqueous solutions of ascorbic acid (0, 1, 10, 100, 300, 500, and 1,000 μM) were added to 1.8 mL aqueous solutions of R2c@(BSA)_2_ magnetically stirred (final conc.: 3 μM), and fluorescence was measured over time. Similar measurements were obtained by adding 1 mL bovine or human sera supplemented with ascorbic acid (0, 30, 50, and 70 μM) to 1 mL aqueous solutions (pH 3) of 6 μM R2c@(BSA)_2_ (final conc.: 3 μM).

### ***In vivo*and*ex vivo*imaging**

*In vivo* imaging was performed on an IVIS Lumina II system (Xenogen) with a Cy5.5 bandpass filter (excitation wavelength 640 nm and emission wavelength 695–770 nm). Nude mice (Balb/c-nu, female, 8 weeks, n = 8) were fed alfalfa-free diet to minimize autofluorescence from chlorophyll, and were injected with 125 μL phosphate buffered saline containing 118 μM R2c@(BSA)_2_ via the tail vein (0.7 nmol per gram of body weight). In experiments analysing liposomal R2c, mice were injected with 125 μL phosphate-buffered saline containing 152 μM liposomal R2c (1 nmol per gram of body weight). Subsequently, a total dose of 0.23 mg of ascorbic acid per gram of body weight was given to mice by intravenously injecting 50 μL phosphate-buffered saline containing 512 mM ascorbic acid 2 h after initial injection of the fluorescent probe. After *in vivo* imaging, mice were sacrificed, and excised organs were imaged *ex vivo* on the same imaging platform. Concentrations of ascorbic acid in the plasma and liver were also examined using a Vitamin C Assay Kit (SHIMA laboratories Co., Ltd.): 15–20 min after the injection of ascorbic acid (0.23 mg per gram of body weight), the blood was obtained from the vena cava, and the liver was excised from mice. *In vivo* macroscopic features of acute toxicity were not observed after the injection of R2c@(BSA)_2_ into mice: this is consistent with the toxicity test of R2c consisting of low-toxic molecules toward cells^40^.

All experiments involving animals were performed in accordance with relevant guidelines approved by the animal experimentation committee of Katayama Chemical Industries, Co., Ltd. According to the requirements for Biosafety and Animal Ethics, all efforts were made to minimize the number of animals used and their suffering.

### Purity of ascorbic acid

Ascorbic acid (≥98%) was purchased from Kanto Chemical Co. Furthermore, because ascorbic acid can be easily oxidized by molecular oxygen, oxidized forms of ascorbic acid, i.e., dehydroascorbic acid and ascorbate radical, were investigated by using proton nuclear magnetic resonance (NMR, JEOL JNM-ECS400) and electron spin resonance (ESR, JEOL JES-FE2XGS EPR SPECTROMETER), respectively. In addition, the degradation of ascorbic acid was traced by detecting the electronic absorption band at 244 nm. Thus, the purity of ascorbic acid was found to be satisfactory under our experimental conditions.

## Electronic supplementary material

Supplementary information
