# Supplementary material for: In vivo fluorescence bioimaging of ascorbic acid in mice: Development of an efficient probe consisting of phthalocyanine, TEMPO, and albumin
Source: Sci Rep. 2018 Jan 24;8:1560. doi: 10.1038/s41598-018-19762-8 (PMC5784034; doi:10.1038/s41598-018-19762-8)
Supplement: Supplementary file 1 — Supplementary information [file 41598_2018_19762_MOESM1_ESM.pdf]

Supplementary Information

In vivo fluorescence bioimaging of ascorbic acid in mice: Development of an efficient probe consisting of phthalocyanine, TEMPO, and albumin

Takanori Yokoi,[1] Takayuki Otani,[2] Kazuyuki Ishii[1*]

[1]Institute of Industrial Science, The University of Tokyo, 4-6-1 Komaba Meguro-ku, Tokyo 153-8505, Japan. [2]Katayama Chemical Industries, Co., Ltd., 4-1-7 Ina, Minoh city, Osaka 562-0015, Japan

Corresponding author. E-mail: k-ishii@iis.u-tokyo.ac.jp

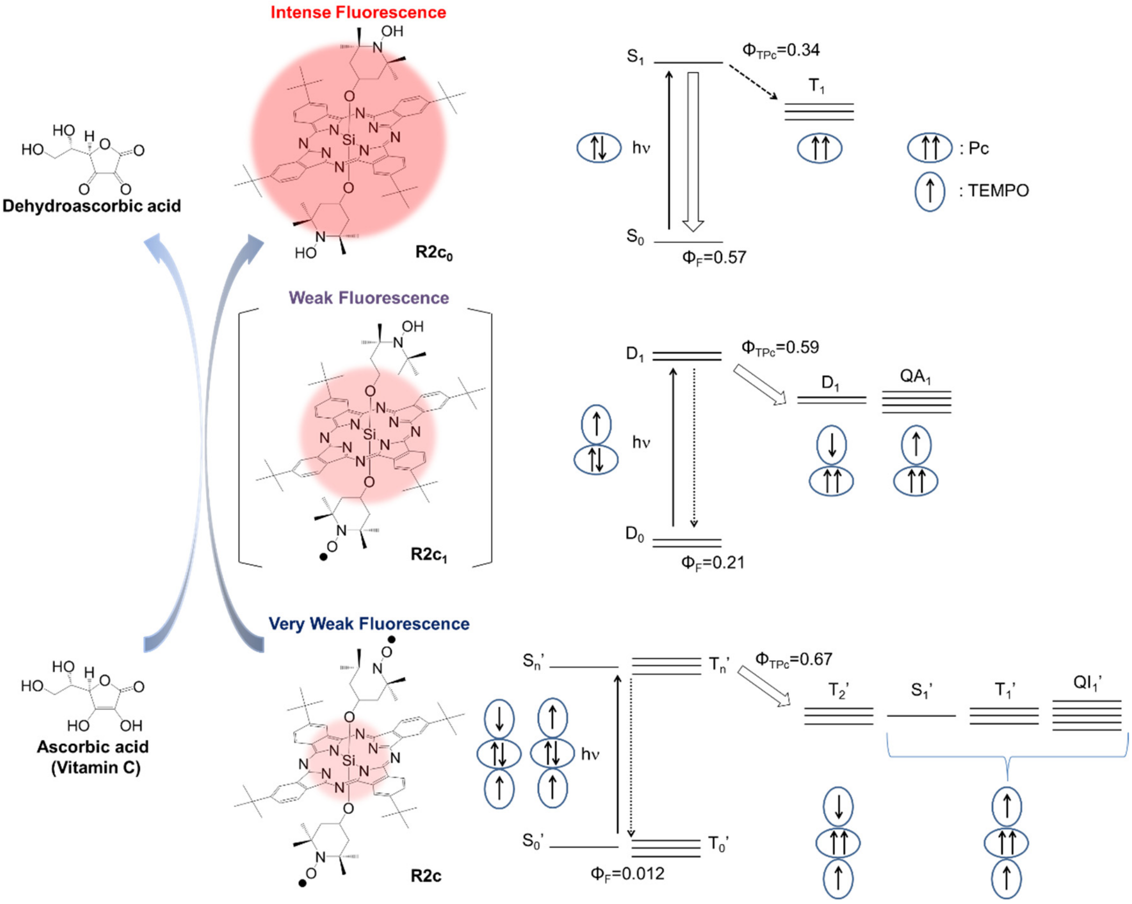

Supplementary Figure S1. Excited-state dynamics of silicon phthalocyanine (SiPc) derivatives, quenching of SiPc fluorescence due to TEMPO radicals, and reaction between R2c and ascorbic acid.

Illustrated are the excited-state properties of (dihydroxy)SiPc (R0), which are fundamentally similar to those of R2c$_0$, the two-electron reduced form of R2c. Ascorbic acid exists as an ascorbate anion when pH is >4.2. The lowest excited singlet (S_1) and triplet (T_1) states, located at ~14500 cm^{-1} and ~9000 cm^{-1}, are nearly derived from the $^1(a_{1u}e_g)$ and $^3(a_{1u}e_g)$ configurations, respectively, with the a_{1u} (π) and e_g (π^*) orbitals denoting the HOMO and LUMO of SiPc. The fluorescence (Φ_F) and triplet (Φ_{TPc}) quantum yields are 0.57 and 0.34, respectively[1-3]. In R2c$_1$, the one-electron reduced form of R2c, the doublet ground (D_0) state and the excited doublet (D_n) state consist of TEMPO in the D_0 state (^{2}TEMPO) and SiPc in the singlet (S_0 or S_1) state (^{1}SiPc or ^{1}SiPc*). On the other hand, the lowest excited doublet (D_1) and quartet (QA_1) states are generated by the interaction between ^{2}TEMPO and SiPc in the T_1 state (^{3}SiPc*). Studies of SiPc linked to one TEMPO radical (R1c) indicated that generation of the $D_n \rightarrow D_1$ transition enhanced the ^{1}SiPc$^* \rightarrow ^3$SiPc* intersystem crossing (Φ_{TPc} = 0.59), and decreased the Φ_F value to 0.21[1-3]. In R2c, the singlet (S_0') and triplet (T_0') ground states are generated by the

interaction between two ^{2}TEMPO radicals. The excited singlet (S_n') and triplet (T_n') states are derived from ^{1}SiPc* and two ^{2}TEMPO radicals. The interactions between ^{3}SiPc* and the two ^{2}TEMPO radicals result in the lowest excited singlet (S_1'), triplet (T_1'), and quintet (QI_1') states, as well as the second lowest excited triplet (T_2') state. Here, the two ^{2}TEMPO radicals exhibit triplet and singlet characters in the T_1' and T_2' states, respectively. As a result of interactions with the two ^{2}TEMPO radicals, the Φ_F value significantly decreased to 0.012, because ^{3}SiPc* (Φ_{TPc} = 0.67) was preferably formed[1-3].

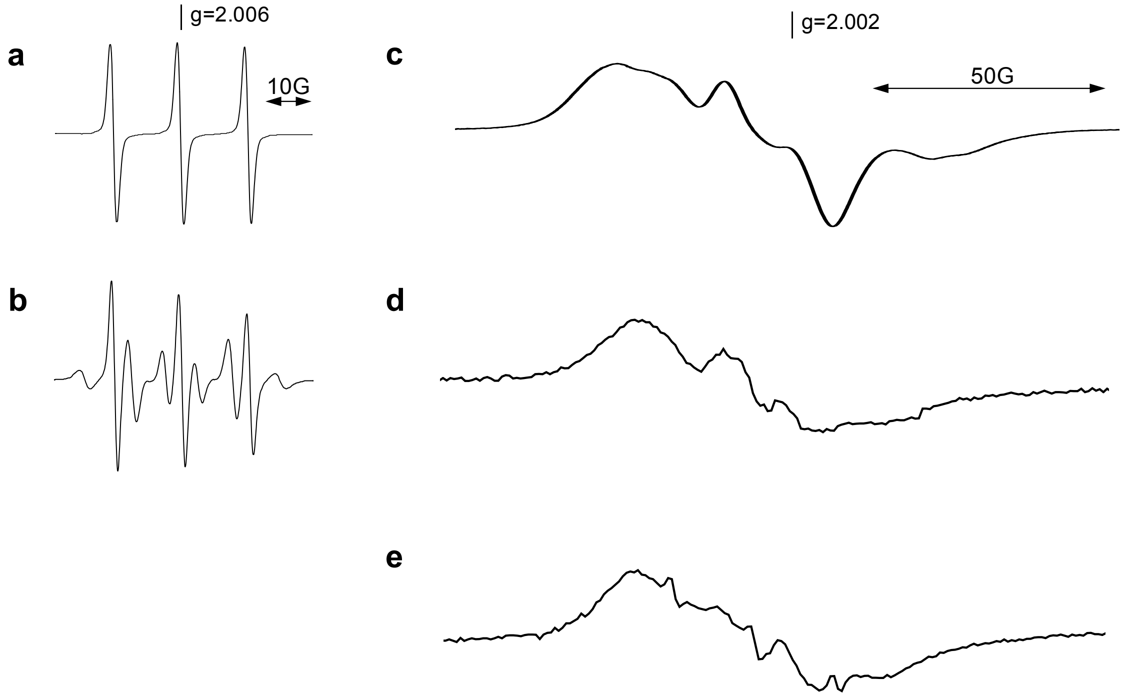

Supplementary Figure S2. Electron spin resonance spectra.

Steady-state electron spin resonance spectra of 4-hydroxy-TEMPO in toluene (a), R2c in toluene (b), R2c in frozen toluene (c), R2c@$(BSA)_2$ in aqueous solution (d), and R2c@TX-100 in aqueous solution (e).

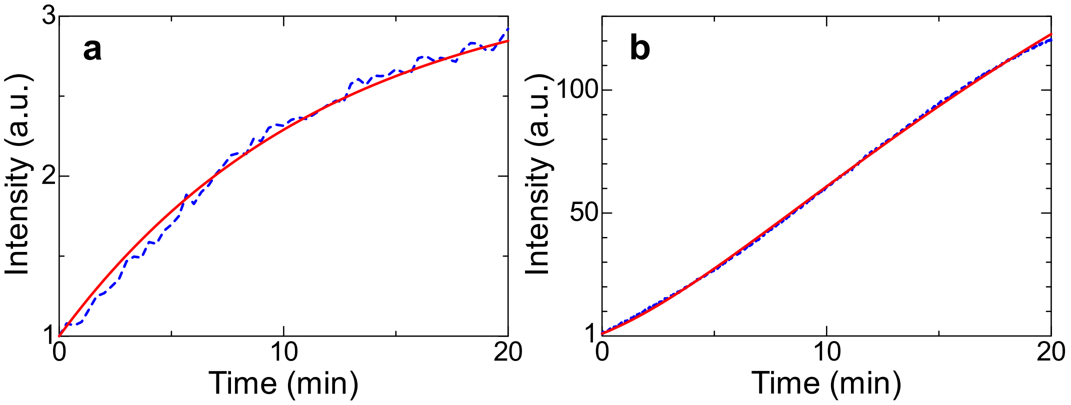

Supplementary Figure S3. Analyses of time courses of fluorescence from liposomal R2c and R2c@$(BSA)_2$.

Time courses (blue dashed lines) of fluorescence from liposomal R2c (a) and R2c@$(BSA)_2$ (b) in aqueous solutions at pH 7.4, which can be analysed, respectively, by a single exponential function (a, red line), and as a function based on consecutive reaction (b, red line).

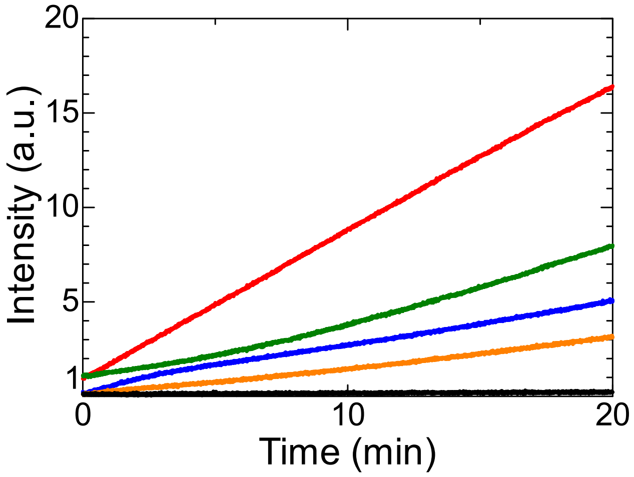

Supplementary Figure S4. pH dependence of fluorescence.

Time courses of R2c@$(BSA)_2$ fluorescence at pH 2 (green), pH 3 (red), pH 4 (blue), pH 5 (orange), and pH 6 (black): the differences between pH 6, 7, and 8 were negligible. Accordingly, pH 3 is the best condition at which to measure R2c@$(BSA)_2$ fluorescence due to ascorbic acid.

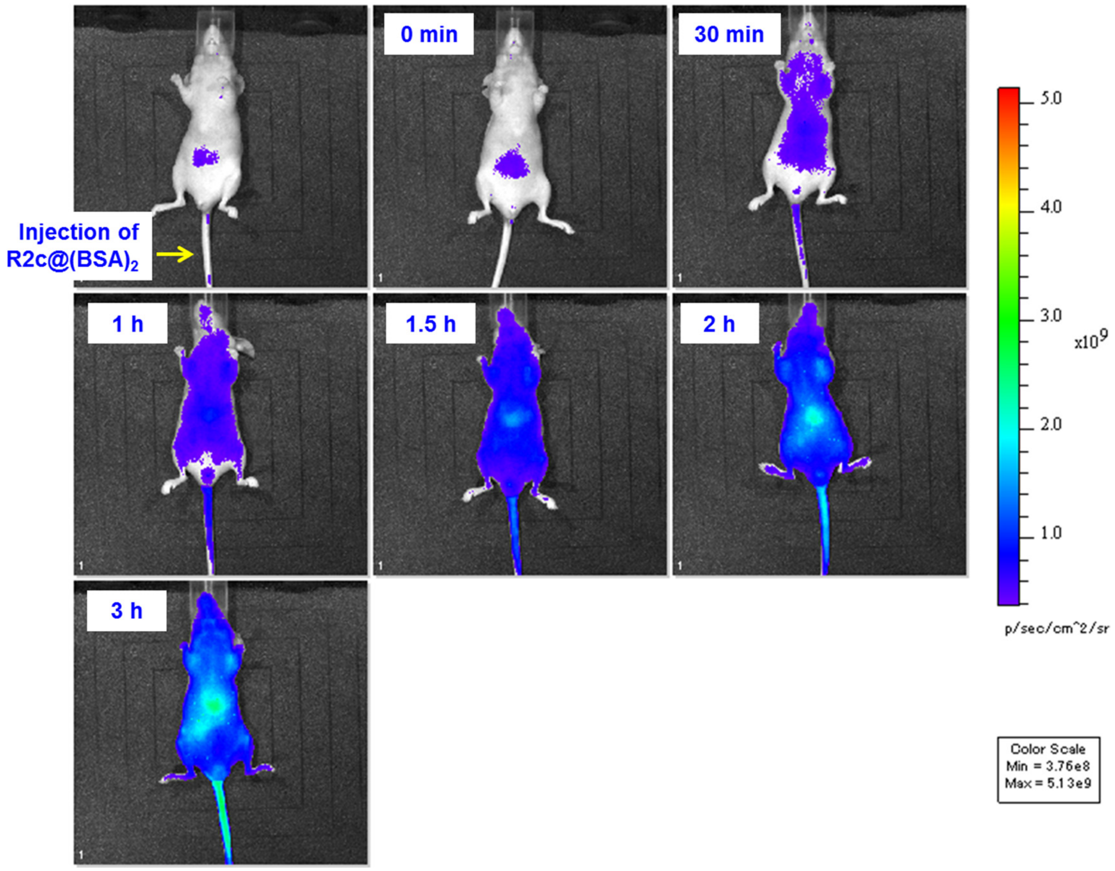

Supplementary Figure S5. Fluorescence imaging of a mouse injected with R2c@$(BSA)_2$.

Representative *in vivo* fluorescence imaging of a mouse injected with R2c@$(BSA)_2$, but not with ascorbic acid. Eventually, fluorescence due to R2c@$(BSA)_2$ was observed throughout the body.

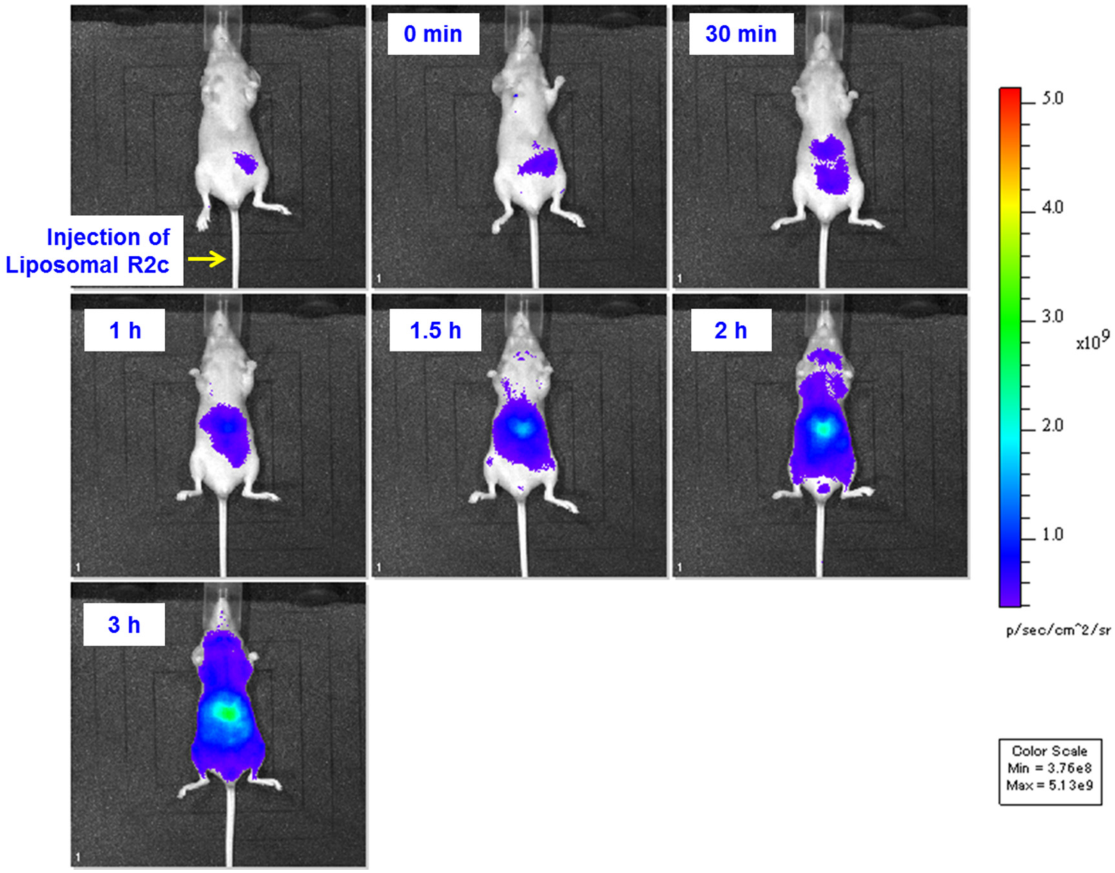

Supplementary Figure S6. Fluorescence imaging of a mouse injected with liposomal R2c.

Representative *in vivo* imaging of a mouse injected with liposomal R2c, but not with ascorbic acid. At the end of experiment, fluorescence was observed throughout the body, but was particularly intense in the liver.

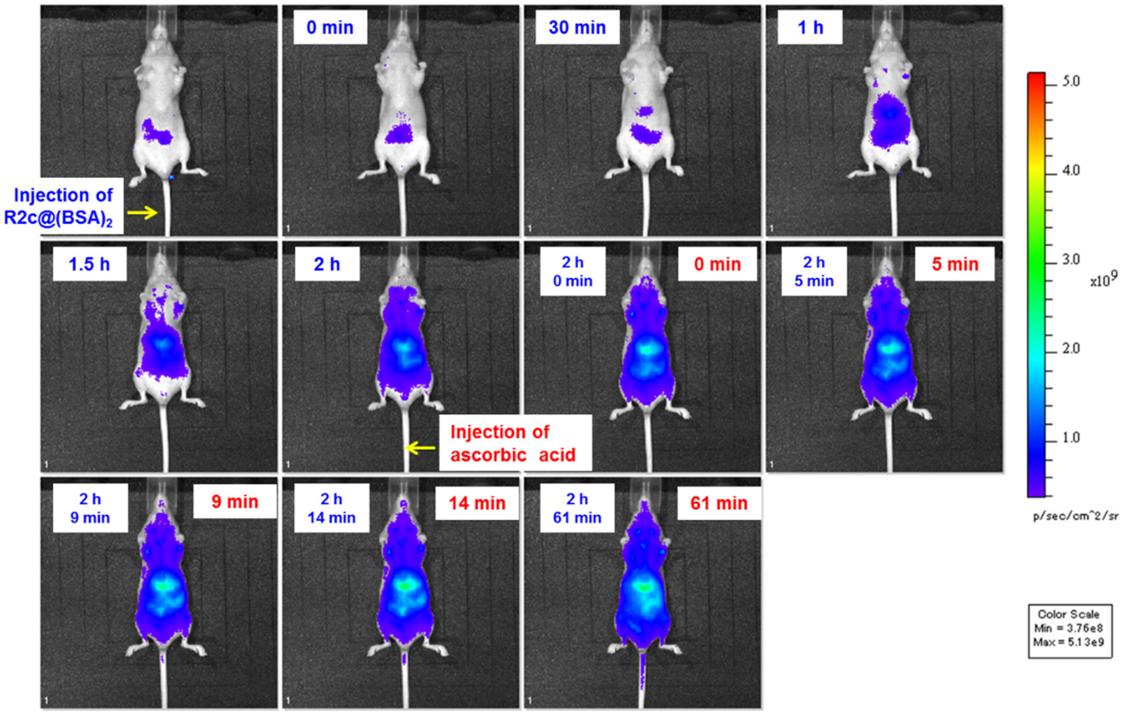

Supplementary Figure S7. Fluorescence imaging of a mouse injected with liposomal R2c and ascorbic acid.

Representative *in vivo* liposomal R2c-based fluorescence imaging of intravenously injected ascorbic acid in a mouse. Time elapsed after R2c@$(BSA)_2$ injection is indicated at the upper left in each image, while that after ascorbic acid injection is shown at the upper right in each image.

Supplementary References

1. Ishii, K., Kubo, K., Sakurada, T., Komori, K. & Sakai, Y. Pthalocyanine-based fluorescence probes for detecting ascorbic acid: phthalocyaninatosilicon covalently linked to TEMPO radicals. *Chem. Commun.* **47**, 4932-4934 (2011).
2. Ishii, K., Hirose, Y., Fujitsuka, H., Ito, O. & Kobayashi, N. Time-resolved EPR, fluorescence, and transient absorption studies on phthalocyaninatosilicon covalently linked to one or two TEMPO radicals. *J. Am. Chem. Soc.* **123**, 702-708 (2001).
3. Ishii, K., Takeuchi, S., Shimizu, S. & Kobayashi, N. A concept for controlling singlet oxygen ($^1\Delta_g$) yields using nitroxide radicals: phtalocyanninatosilicon covalenly linked to nitroxide radicals. *J. Am. Chem. Soc.* **126**, 2082-2088 (2004).
